# A single vector-based strategy for marker-less gene replacement in *Synechocystis* sp. PCC 6803

**DOI:** 10.1186/1475-2859-13-4

**Published:** 2014-01-08

**Authors:** Stefania Viola, Thilo Rühle, Dario Leister

**Affiliations:** 1 Department Biology I, Ludwig-Maximilians-Universität München, Großhaderner Str. 2, Planegg, Martinsried D-82152, Germany

**Keywords:** Genetic engineering, Gene replacement, Homologous recombination, *Synechocystis*

## Abstract

**Background:**

The cyanobacterium *Synechocystis* sp. PCC 6803 is widely used for research on photosynthesis and circadian rhythms, and also finds application in sustainable biotechnologies. *Synechocystis* is naturally transformable and undergoes homologous recombination, which enables the development of a variety of tools for genetic and genomic manipulations. To generate multiple gene deletions and/or replacements, marker-less manipulation methods based on counter-selection are generally employed. Currently available methods require two transformation steps with different DNA plasmids.

**Results:**

In this study, we present a marker-less gene deletion and replacement strategy in *Synechocystis* sp. PCC 6803 which needs only a single transformation step. The method utilizes an *nptI-sacB* double selection cassette and exploits the ability of the cyanobacterium to undergo two successive genomic recombination events via double and single crossing-over upon application of appropriate selective procedures.

**Conclusions:**

By reducing the number of cloning steps, this strategy will facilitate gene manipulation, gain-of-function studies, and automated screening of mutants.

## Background

Cyanobacteria, also known as blue-green algae, are Gram-negative, photosynthetic prokaryotes. Moreover, they are the evolutionary ancestors of plastids [1] and the only prokaryotes that exhibit a classical circadian clock mechanism [2]. Thanks to their ability to adapt to extreme environmental conditions, cyanobacteria are widely distributed. These features, and their recent exploitation for biotechnological purposes [3,4], explain why cyanobacteria have been extensively studied. Over the past few decades, numerous cyanobacterial genomes have been sequenced (http://genome.microbedb.jp/cyanobase) and many molecular tools for their genetic manipulation have been developed. *Synechocystis* sp*.* PCC 6803 (hereafter designated as *Synechocystis*) is an excellent model organism, because it has a small (3.6 Mb), sequenced genome [5], is capable of spontaneous uptake of exogenous DNA and can integrate it via homologous recombination into its genome. In addition, a spontaneous glucose-tolerant mutant is available [6] that can grow heterotrophically in the presence of glucose and is thus useful for the study of oxygenic photosynthesis. Photosynthetic *Synechocystis* mutants have been extensively used to study photosystems I (PSI) [7,8] and II (PSII) [9,10] and to investigate the functionality of photosynthesis-related proteins from higher plants in cyanobacteria [11,12]. All these studies have employed gene deletions or replacements.

The classical means of deleting a target gene or inserting exogenous genetic material into the *Synechocystis* genome via homologous recombination involves the use of a resistance marker [9,13]. In this approach, the insertion cassette (marker plus exogenous DNA) is flanked by sequences homologous to the targeted site in the genome, and the whole segment is cloned into a suicide vector, which is unable to replicate in the host cell. After integration of the insertion cassette into the host genome, the resistance-mediating marker allows for positive selection of the mutant cells in which integration has occurred. However, to perform additional genomic modifications, one must use other resistance markers. Consequently, the number of available markers restricts the number of genetic manipulations. To overcome this limitation, so called marker-less strategies have been developed, which allow for the removal of the integrated marker. The first marker-less method was established in Gram-negative bacteria using the *nptI-sacB* double selection cassette [14]. Here, the *nptI* gene confers resistance to the antibiotic kanamycin, while expression of the *sacB* gene from *Bacillus subtilis*[15,16] is toxic to Gram-negative bacteria grown on sucrose-containing media. SacB is a levansucrase that hydrolyzes sucrose, and utilizes the fructose unit for the production of levans [17]. The polymers are toxic to Gram-negative bacteria, and cells harbouring the *sacB* gene die when grown in presence of 5% sucrose, although the underlying mechanism is not fully understood. In cyanobacteria, the *nptI-sacB* cartridge was first used to establish marker-less gene replacement in *Anabaena sp.* PCC 7120 [18]. Gene replacement requires two homologous recombination events, each of which requires a suicide vector and a bacterial transformation step (see Additional file 1). 1) The target genomic sequence is replaced by the double-selection cassette, and the mutants in which the homologous recombination has taken place are positively selected by their ability to grow in the presence of the antibiotic. 2) A second transformation and homologous recombination event leads to the excision of the *nptI-sacB* selection cartridge and its concomitant replacement by the exogenous sequence. The desired mutants can be selected on the basis of their resistance to sucrose and sensitivity to kanamycin. An alternative version of double selection was developed for gene replacement in *Synechococcus elongatus* PCC 7942 [19]. In this approach, the background strain carries a mutated form of the *rps12* gene (which codes for subunit S12 of the 30S ribosome) which confers resistance to spectinomycin [20,21]. Since the mutation is recessive, the double selection cassette carries a kanamycin resistance gene and - as an alternative negative selection marker - a wild-type copy of *rps12*, which results in a dominant spectinomycin-sensitive phenotype. A drawback of the *rps12* marker system is the need to perform all multiple replacements in the *rps12* mutant background, which introduces the need of an additional manipulation step. A general weakness of the marker-less gene replacement methods described above is that two time-consuming transformation events and cloning of two different suicide vectors are required. Alternative double recombination strategies have been developed that rely on initial integration of the whole vector into the target genomic sequence via single crossing-over, as in the case of allelic exchange [22,23]. However, in this case, the second recombination, leading to the excision of the integrated vector, gives rise to both (reverted) wild-type cells and bacteria that retain the desired mutation, so that this population of recombinants must be screened by PCR.

The aim of the present study was to develop a time-saving strategy for marker-less gene replacement in *Synechocystis* which requires only a single vector and a single bacterial transformation event. The strategy finally chosen leads to a population which consists exclusively of mutants. Therefore, the method offers a number of technical advantages over the classical approaches and is applicable to other bacterial species capable of undergoing gene transfer, irrespective of the selective markers employed.

## Results

### Design of the single-step double-recombination vectors

**Figure 1:**
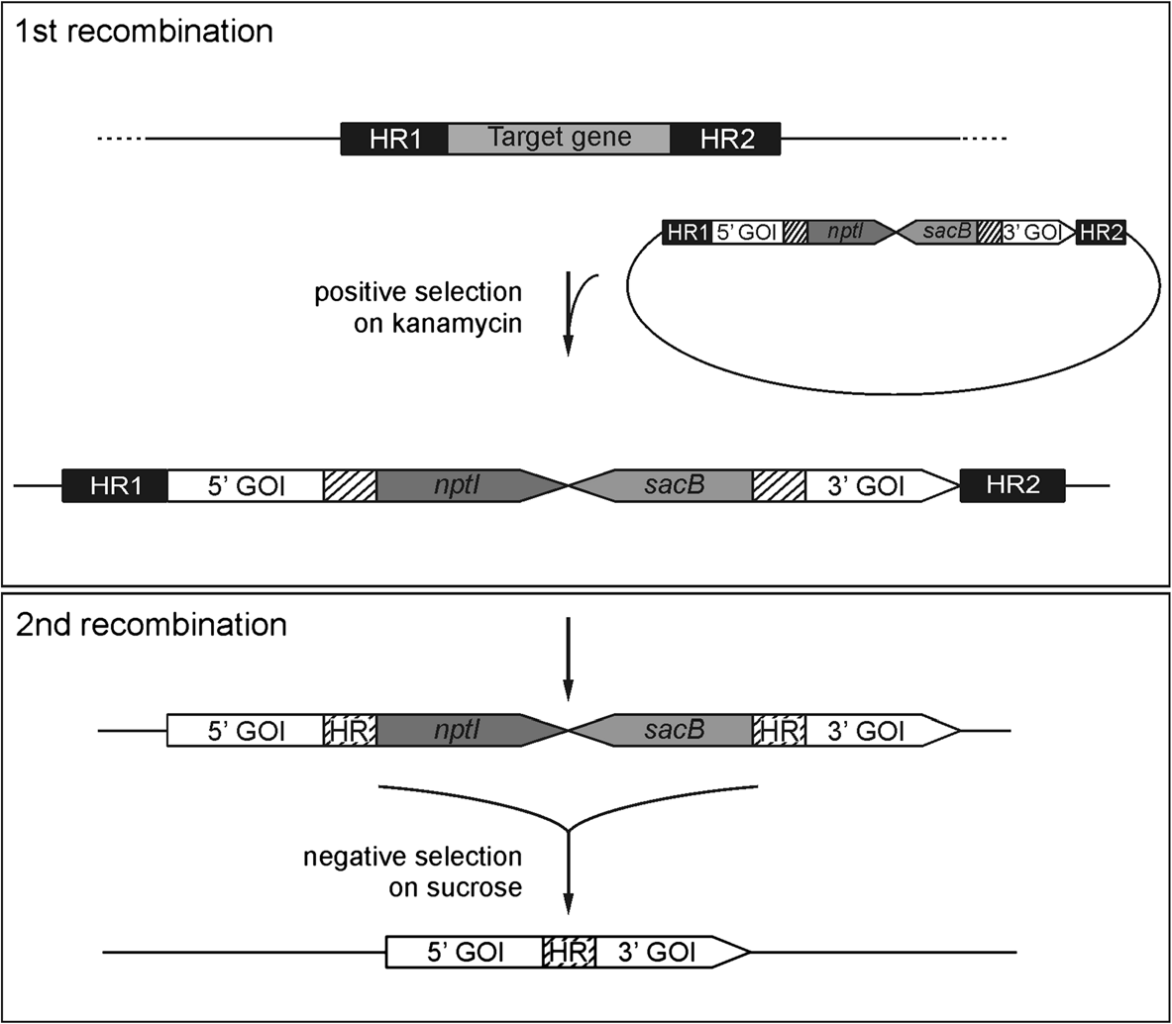
**Schematic depiction of the single-step double recombination strategy.** The first recombination step (upper panel), involving a double crossover between the homologous regions HR1 and HR2 of the vector and the genomic target sequence, leads to genomic integration of the construct. Note that the integrated gene of interest (GOI) is split into two parts, 5’ and 3’, the sequences of which partially overlap (shaded box). The 5’ and 3’ GOI segments are separated from each other by the *nptI-sacB* selection cassette, which renders the first recombinant mutants resistant to kanamycin and sensitive to sucrose. After complete segregation of the replacement under positive selection in the presence of kanamycin, which ensures the total elimination of the endogenous target gene function, release of the selective pressure allows the second recombination to take place (lower panel). In this step, a crossover involving the overlapping regions of the split GOI leads to the excision of the *nptI-sacB* cassette. Negative selection on sucrose yields colonies that have lost the entire *sacB* marker and carry the intact, functional GOI in place of the endogenous target gene.

A vector designed for a single-step double-recombination approach (see Figure 1) must include several essential elements: 1. an origin of replication for *E. coli* that is non-functional in *Synechocystis*; 2. flanking sequences homologous to *Synechocystis* chromosomal sites to allow stable integration of the constructs into the cyanobacterial genome (designated as HR1 and HR2); 3. the *nptI-sacB* double selection cassette that allows both positive and negative selection of the *Synechocystis* recombinants; 4. the exogenous gene of interest (GOI), split into two, partially overlapping (shaded box) segments (5’ GOI and 3’ GOI) that are separated by the *nptI-sacB* cassette; 5. a *Synechocystis* promoter for expression of the introduced recombinant gene(s).

When *Synechocystis* cells are transformed with such a vector, the initial recombination event takes place (Figure 1, upper panel) via a double crossover event between the two flanks of the construct, HR1 and HR2, and the corresponding homologous sequences present in the cyanobacterial genome. Mutants that have integrated the construct in this way contain the *nptI-sacB* cassette in the target locus and can be positively selected on kanamycin. These recombinants are resistant to the antibiotic but sensitive to sucrose. In these mutants the GOI is not functional, because its sequence is disrupted by the *nptI-sacB* cassette. Moreover, complete segregation of the first-round [“prim(ary)”] recombinants leads to loss of the endogenous target gene. The loss of the target gene also proves that integration of the construct happened via a double crossover event on HR1 and HR2 and not via a single crossover event involving only one flanking sequence. After complete segregation, the positive selective pressure is released and the mutants are grown in the absence of selection in order to allow the second recombination event to occur (Figure 1, lower panel), without the need for a further transformation step. The second crossover event occurs between the internal overlapping sequences of the foreign DNA and leads to the excision of the double selection cassette, restoring the integrity of the introduced gene(s). Mutants in which the second recombination is incomplete can be efficiently counter-selected on sucrose, because only those recombinants that have lost the whole *nptI-sacB* cassette will survive. In consequence, in the population generated by the second round of recombination [“sec(ondary) recombinants”], the target gene is replaced by the transgenic DNA of choice and no selection marker remains in the genome.

### Confirmation of the strategy: 1. Introduction of the luciferase reporter system

**Figure 2:**
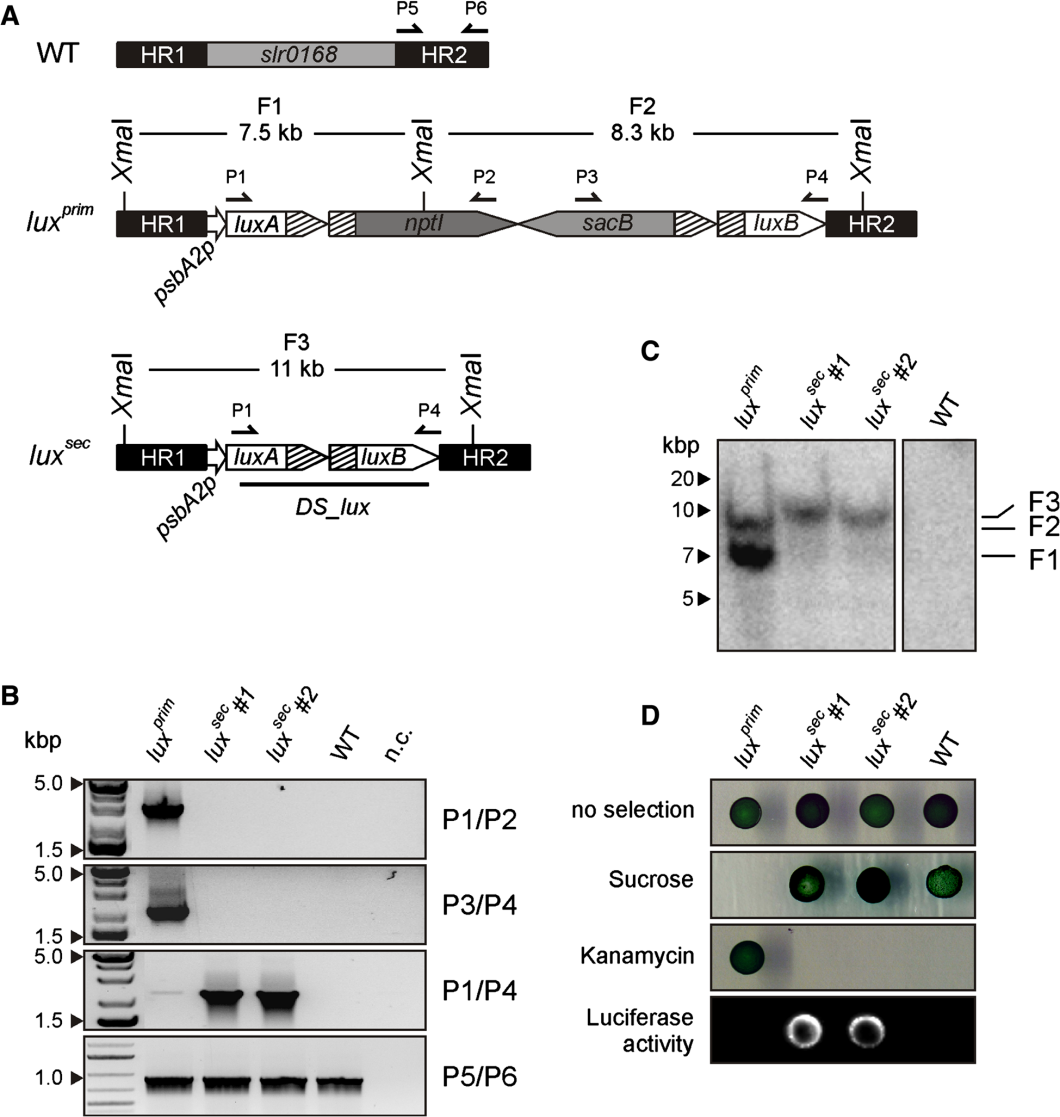
**Analysis of the *lux *strains. A.** Schematic depiction of the mutant strains after the first and second rounds of recombination. In *lux*^*prim*^, the *nptI-sacB* cassette interrupts the *luxB* gene. In *lux*^*sec*^, loss of the cassette leads to the reconstitution of the entire *luxAB* operon under the regulation of the *psbA2* promoter. Annealing sites of the primers used for genotyping (P1-6) and of the *DS_lux* probe used for Southern hybridization as well as the positions of the *XmaI* restriction sites are indicated. **B.** Complete segregation of the *Synechocystis lux* strains generated by the genetic manipulations represented in A. Genotyping PCR was performed on five independent *lux*^*prim*^ first-round recombinants and, for each of them, two second-round recombinants. Note that *lux*^*prim*^#2-5 behaved like *lux*^*prim*^#1. Expected sizes of the amplicons generated by the used primer pairs were: P1/P2, 2.7 kbps; P3/P4, 2.3 kbps; P1/P4, 2.3 kbps; P5/P6, 1 kbps. Negative control (n.c.) was included. **C.** Southern analysis of genomic DNA from *Synechocystis* WT and *lux* mutants. The *XmaI* restriction map of the *slr0168* genomic region in the analyzed strains and the probe used for hybridization are shown in A. Five μg of DNA were loaded per lane. Genomic fragments F1 (7.5 kbps) and F2 (8.3 kbps) were detected in *lux*^*prim*^, the fragment F3 (11 kbps) in the *lux*^*sec*^ strains. Note that *lux*^*prim*^#2-5 behaved like *lux*^*prim*^#1. The lane corresponding to WT is also shown. **D.** Drop test of *lux* mutants on selective media. Liquid cultures (OD_730_: 0.4) were washed with BG11 without glucose and spotted (15 μl each) onto BG11 medium containing either 100 μg/ml kanamycin or 5% sucrose or no supplement. Colonies from the non-selective plate were incubated with 1 mM decanal and luminescence was determined to quantify luciferase activity.

WT *Synechocystis* cells were transformed with the vector pDSlux (Figure 2A). The mutants obtained after the first round of recombination, *lux*^*prim*^, harboured an interrupted *luxAB* operon inserted in the *Synechocystis slr0168* ORF. After complete segregation, all the analyzed strains were knockouts for the *slr0168* target locus, thus confirming the correct double crossover event in the first recombination step. In the mutants obtained after the second recombination round, named *lux*^*sec*^, excision of the double selection cassette led to the reconstitution of the intact *luxAB* operon under the control of P_*psbA2*_.

The frequencies of the first and second recombination events were calculated for *lux* mutants. Ten independent *lux*^*prim*^ strains obtained after the first recombination were selected and allowed to undergo a second recombination. The transformation frequency was 4 × 10^-5^, which is comparable to the values reported in the literature [24]. The frequency of the second recombination varied between 2 × 10^-5^ and 1 × 10^-7^ (Table 1), with an average value of 9 × 10^-6^, revealing that it occurs with a frequency about ten times lower than the first. All the analyzed second-round recombinants were not able to grow in the presence of kanamycin, confirming that the sucrose resistance was due to loss of the double selection cassette and not to inactivation of the *sacB* gene by spontaneous mutations. Five of the original ten independent *lux*^*prim*^ first recombinants and two *lux*^*sec*^ second recombinants for each of the five *lux*^*prim*^ clones were selected for further analyses.

**Table 1:** **Frequency of the second recombination event in independent *lux***^***prim ***^**strains**

***lux***^***sec***^	**#1**	**#2**	**#3**	**#4**	**#5**	**#6**	**#7**	**#8**	**#9**	**#10**
	7×10^-6^	5×10^-7^	7×10^-6^	4×10^-6^	7×10^-6^	1×10^-7^	2×10^-6^	2×10^-5^	2×10^-5^	2×10^-5^

To confirm the correct segregation of the expected genetic variants in these mutants, genomic PCR was performed using three primer pairs. In all five *lux*^*prim*^ mutants, but in none of the *lux*^*sec*^ strains, the primer pairs P1 + P2 and P3 + P4 generated amplicons (2.7 and 2.3 kbps, respectively) spanning the 5’ or 3’ regions respectively of the *nptI-sacB* cassette and the flanking segment of the interrupted *luxAB* operon. When combined, primers P1 and P3 amplified the reconstituted *luxAB* operon, generating a PCR product of 2.3 kbps that was detectable only in the *lux*^*sec*^ second recombinants. The *slr0168* downstream region (HR2, ~1 kbps), used as a positive control, could be amplified in all the samples using the primer pair P5 + P6 (Figure 2B).

Gene replacement via homologous recombination is sometimes prone to error, and may result in non-homologous DNA integration into the genome, thus generating mutants with multiple or non-targeted genomic insertions. However, when Southern blots bearing genomic *XmaI* digests from WT and the selected *lux* mutants were probed with a fragment of the *luxAB* recombinant operon (Figure 2C), two labelled bands (F1 and F2, with 7.5 and 8.3 kbps respectively) corresponding to the split *luxAB* operon were found in the *lux*^*prim*^ mutants, while the detection of a single band of 11 kbps (F3) in the *lux*^*sec*^ recombinants confirmed the presence of the intact operon. The absence of any signal of unexpected size indicated that homologous recombination had occurred at the correct target locus in all cases.

In order to phenotypically characterize the *lux* mutants, they were grown on various BG11-based media (Figure 2D). When spotted on BG11 plates with 5 mM glucose and cultured without selection pressure, all *lux*^*prim*^ and *lux*^*sec*^ mutant strains grew normally. The *lux*^*prim*^ cells resulting from the first round of recombinants grew in the presence of kanamycin. Furthermore, the presence of 5% sucrose in the medium was lethal for the *lux*^*prim*^ strains, confirming the functionality of the negative selection marker. Conversely, all the *lux*^*sec*^ strains were sensitive to kanamycin, as they had lost the resistance marker, and grew on BG11 supplemented with 5% sucrose because they no longer harboured the *sacB* gene. None of the strains could grow on BG11 supplemented with both kanamycin and sucrose, as expected. This confirms that no integration of the whole vector, also bearing a kanamycin resistance gene on the backbone, via a single crossover event, took place during the first recombination step.

The *luxAB* operon codes for two proteins that are both required for the expression of luminescence, a phenotype not naturally present in *Synechocystis*. In *lux*^*prim*^ mutants the full-length *luxB* gene is not fused to a functional promoter; therefore, the LuxB subunit of the luciferase cannot be synthesized. The second recombination (in *lux*^*sec*^ mutants) is expected to reconstitute the entire *luxAB* operon under the regulation of P_*psbA2*_, thus allowing the expression and assembly of the functional enzyme. To confirm this, all the spotted strains were tested for luminescence (Figure 2B, lower panel). Strong luciferase activity was detectable only in the *lux*^*sec*^ strains carrying the reconstituted *luxAB* operon, and almost absent in all the *lux*^*prim*^ mutants. A faintly luminescent background was always detectable in the first-round recombinants, possibly due to read-through transcription of *luxB* from the *nptI* promoter. To better quantify the luciferase activity, light emission was measured for the ten *lux*^*prim*^ strains used for second recombination and ten *lux*^*sec*^ independent recombinants for each of them (Table 2). The luminescence values relative to the untransformed WT *Synechocystis* strain clearly indicated a gain of function in all the *lux*^*sec*^ strains, albeit with a certain inter-strain variability. The level of luciferase activity in the *lux*^*prim*^ transformants ranged between 1.5- and 2.5-fold higher than in the WT, while in the second-round recombinants it was at least ten times higher than in WT.

**Table 2:** **Luciferase activity relative to OD**_**730 **_**in *Synechocystis lux***^***prim***^**and *lux***^***sec ***^**mutants**

	_**#**_**1**	**#2**	**#3**	**#4**	**#5**	**#6**	**#7**	**#8**	**#9**	**#10**
***lux***^***prim***^	2.6 ± 1.4	2.3 ± 0.4	1.8 ± 0.4	2 ± 1.2	2.1 ± 0.7	1.8 ± 0.5	2.2 ± 1.1	1.7 ± 0.6	1.5 ± 0.8	1.5 ± 1
***lux***^***sec***^ (1–10)	17.3 ± 3.9	61.8 ± 11.3	16.6 ± 3.6	24.1 ± 6.5	15.3 ± 5.1	23.2 ± 9.6	22.7 ± 9.6	10.4 ± 3.2	10.6 ± 2.9	13.1 ± 3.8

For each strain, the first recombinant and ten independent second recombinants were analyzed. Each suspension was measured in duplicate and the assay was repeated twice with independently grown cultures. Average luminescence values of the mutants are relative to background signal in the untransformed WT *Synechocystis* strain, which value was set to 1.

### Confirmation of the strategy: 2. The *psaA* gene

**Figure 3:**
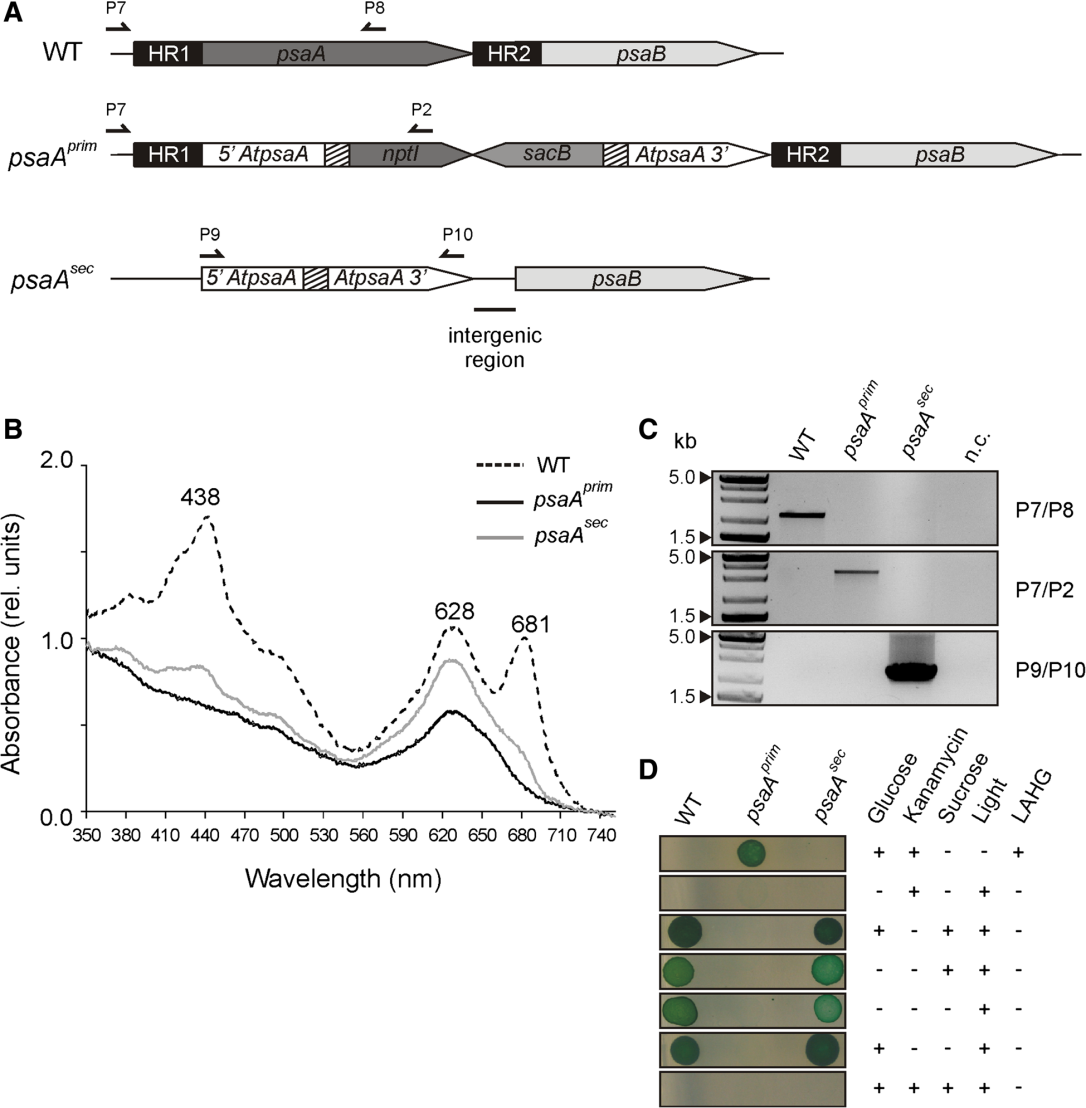
**Analysis of the *psaA *strains. A.** Schematic depiction of mutant strains after the first and second rounds of recombination following transformation with the *psaA* construct. The *psaA* construct is integrated in the *Synechocystis psaA* gene. In *psaA*^*prim*^ the *nptI-sacB* cassette interrupts the *At psaA* gene. In *psaA*^*sec*^, loss of the cassette leads to the reconstitution of the entire *At psaA* under the control of the native cyanobacterial promoter. Positions of the primers used for genotyping (P2, P7-10) are indicated. **B.***In vivo* absorption spectra of WT, *psaA*^*prim*^ and *psaA*^*sec*^*Synechocystis* strains. The peaks at 438 and 681 nm correspond to the maxima of Chl *a* absorption, the peak at 628 nm corresponds to the absorption maximum of PC. The spectra were normalized to the absorbance at 730 nm. **C.** Complete segregation of the generated *Synechocystis psaA* strains, as demonstrated by PCR analysis. Primer positions are given in A. Expected sizes of the amplicons generated by the used primer pairs were: P7/P8, 2 kbps; P7/P2, 3 kbps; P9/P10, 2.3 kbps. Negative control (n.c.) was included. **D.** Drop test of WT, *psaA*^*prim*^ and *psaA*^*sec*^*Synechocystis* strains on selective media and under different light conditions. Liquid cultures at OD_730_ of 0.4 were washed with BG11 without glucose and spotted (15 μl each) on non-selective BG11 medium or on BG11 containing 100 μg/ml kanamycin or 5% sucrose. When tested for autotrophic growth, cells were grown in continuous light at 30 μmol photons m^-2^ s^-1^ on BG11 without glucose. When grown in Light Activated Heterotrophic Growth (LAHG) conditions, the cells were incubated in the dark on BG11 supplemented with 5 mM glucose, and exposed to light for 5 min every 24 hours.

As photosynthesis is one of the core research areas in which cyanobacteria are extensively used, the same strategy was used to replace the *Synechocystis psaA* (*Syn psaA*) gene with the corresponding homolog from the green plant *Arabidopsis thaliana* (*At psaA*). The *psaA* gene codes for PsaA, a core subunit of PSI [25], and in both cyanobacteria and plants it is part of the *psaA/psaB* operon. The two genes are regulated by the P_*psaA*_ promoter located upstream of *psaA*, and are separated by an intergenic region of 246 base pairs. By transforming the glucose-tolerant WT *Synechocystis* strain with the vector pDSpsaA, analogously to the Lux experiment described above, knockout (*psaA*^*prim*^) and replacement (*psaA*^*sec*^) mutants were subsequently obtained (Figure 3A). The first recombination event yielded colonies in which the integrated construct replaced the endogenous *psaA* gene, generating the knockout line *psaA*^*prim*^. The replacement in *psaA*^*sec*^ affected only the *psaA* coding sequence and left the surrounding genomic regions unchanged, thus retaining all the endogenous regulatory elements intact. In the *psaA*^*prim*^ mutant, the integrated *At psaA* gene was split into two parts and therefore non-functional. Moreover, also expression of PsaB can be expected to be impaired, because (i) the construct integrated between the common promoter and the *psaB* gene, and (ii) disruption of *psaA* leads anyhow to the complete lack of any functional PSI, as both PsaA and PsaB cannot accumulate [26]. In consequence, the *psaA*^*prim*^ strain showed a severe phenotype, since it lacks the PsaA and PsaB proteins, the loss of which prevents the accumulation of any PSI complex [26] and accounts for the turquoise-blue colour of the cells, their high light sensitivity and inability to perform photosynthesis. The absorption spectrum of the mutants showed a complete depletion of chlorophyll *a* (Chl) with the only presence of the phycobilisome chromophore phycocyanin (PC), responsible for the “blue” phenotype (Figure 3B). After complete segregation of the first replacement, removal of the selective pressure led to the second recombination event and subsequent negative selection on sucrose-containing medium yielded colonies that had lost the *nptI-sacB* cassette and carried the reconstituted *At psaA* gene in place of the endogenous one. These *psaA*^*sec*^ mutants were also bluish, having a drastically reduced Chl/PC ratio with respect to the wild-type, but they were able to accumulate more chlorophyll than the *psaA*^*prim*^ strain (Figure 3B). The second-round recombinants were not as light sensitive as the first-round recombinants, and they could grow under normal light conditions.

To confirm correct segregation of the introduced sequences in these mutants, genomic PCR was performed using three primer pairs (Figure 3C). The region (2 kbps) spanning the upstream homologous region used for integration of the construct (HR1) and the endogenous *psaA* gene could be amplified with the primer pair P7 + P8 only in WT, confirming that in both mutant strains the knockout was complete. In *psaA*^*prim*^, but not in WT and in the *psaA*^*sec*^ mutant, the primer pair P7 + P2 generated an amplicon (3 kbps) spanning HR1, the flanking 5’ *At psaA* segments and the 5’ region of the *nptI-sacB* cassette. Primers P9 and P10 amplified the fully reconstituted *psaA* gene from *A. thaliana*, generating a PCR product of 2.2 kbps that was detectable only in the *psaA*^*sec*^ recombinants.

Phenotypic analysis was performed by spotting strains onto different BG11-based media (Figure 3D). The *psaA*^*prim*^ mutant was able to grow on BG11 supplemented with kanamycin, as it harboured the *nptI* resistance gene but, being PSI-deficient, it only grew heterotrophically in presence of 5 mM glucose and under Light Activated Heterotrophic Growth (LAHG) conditions. In LAHG, bacteria are grown heterotrophically in the dark except for a single short exposure to dim light every 24 h, which was suggested to promote the cell division or the progression through the cell cycle [27]. In contrast, under continuous bright light and in the absence of glucose growth of *psaA*^*prim*^ was completely inhibited. The *psaA*^*sec*^ recombinant was unable to grow on kanamycin but could grow on BG11 containing sucrose, having lost the *nptI-sacB* cassette, whereas the presence of 5% sucrose was lethal to the knockout strain. None of the mutants, as expected, was viable in presence of both kanamycin and 5% sucrose. Interestingly, *psaA*^*sec*^ was not light sensitive and was able to grow photoautotrophically on BG11 agar plates containing no glucose. From these results it can be concluded that the At PsaA protein can partially functionally replace the *Synechocystis* protein*,* at least to the extent of restoring light tolerance and the ability to grow photoautotrophically.

## Discussion

Targeted gene and genome manipulation via homologous recombination in bacteria relies on the use of marker genes. In marker-less gene replacement, a negative and a positive selection marker are employed, in a process that normally involves two transformation steps with two DNA suicide vectors. In this work, we developed an alternative strategy for marker-less gene replacement in *Synechocystis*, based on the use of a single plasmid and a single transformation step. The technique was tested by inserting the *luxAB* operon from *Vibrio fischeri* into the neutral receptor site *slr0168*. To this end the wild-type strain was transformed with the vector pDSlux and the transformation efficiency with this integration vector was in the range of 10^-5^, while the average frequency of the second recombination event was 10^-6^ (see Table 1). This ten-fold difference could be due to the fact that the first recombination event involves a double crossover between the genomic DNA and the plasmid vector, whereas the second recombination presumably involves a single intra-genomic crossover [23]. Thus, a difference in efficiency between these two recombination mechanisms could explain the results obtained. Secondary recombinants were easily obtained with 509 bps (*psaA*) and 1055 bps (*luxAB*) of homologous regions; however, the exact impact of the length of the homologous region on recombination frequency remains to be determined. In addition, other factors like position and relative concentration of the homology regions might contribute to the difference in efficiency [28].

In comparison with standard procedures, the single-step marker-less gene replacement strategy described here has several advantages. The requirement of only one vector for each gene replacement makes the cloning procedure faster and more facile, especially when one needs to sequentially replace many genes. Our single-step approach merely requires alternative selective growth conditions in addition to the transformation step, although this flexibility requires that the constructs must be assembled from numerous DNA fragments - six (plus the vector backbone) in the case of pDSlux, for instance. However, the increased complexity of the plasmid vectors required can easily be accommodated by using large-scale modular cloning technologies like the Golden Gate [29], the Gibson [30] or the BioBrick [31] assembly systems. Another issue that can arise during gene manipulation concerns the cloning of genes that are toxic to *E. coli*. Also in this regard, our strategy is advantageous, because the interruption of the GOI sequence by the intervening *nptI-sacB* cassette inactivates it, thus avoiding the problem of toxicity. Only the second recombination in the *Synechocystis* host cell reconstitutes both the gene sequence and function. Also for studies of biological functions for which two genes are necessary, the single-step gene replacement strategy can constitute a suitable experimental tool, as shown for the *luxAB* operon. In the *lux*^*prim*^ mutants, interruption of this operon by the double selection cassette leads to impairment of the luciferase activity, as *luxB* cannot be transcribed and therefore the heterodimeric enzyme cannot be assembled. Only after removal of the cassette by the second recombination event is the integrity of the dicistronic *luxAB*, and with it the luciferase activity restored, as shown in Figure 2A and Table 2. Some background luminescence was observed in the *lux*^*prim*^ recombinants, which could be due to read-through transcription of *luxB* from the *nptI* promoter. Thus, by using only one vector and performing a single transformation step it was possible to show that both *luxA* and *luxB* are necessary for luciferase function, as previously described [32,33]. These results confirm that, when our strategy is used to analyse a gain of function, the new function can be expected to arise only in the second recombinants and not in the first mutant strains, which can therefore serve as ideal controls.

The single-step strategy was also employed to replace the *psaA* gene from *Synechocystis* with the homolog from *Arabidopsis thaliana*, which resides in the plastid genome of the plant. The PsaA subunit, together with PsaB, constitutes the dimeric core of PSI, and is highly conserved among photosynthetic organisms from cyanobacteria to flowering plants [34]. PsaA is an essential component of PSI and is necessary for its assembly, accumulation and function, and therefore essential for photoautotrophic growth of both plants and cyanobacteria. Knockout of the *psaA* gene is lethal to higher plants, while genetic inactivation of the PSI reaction centre in *Synechocystis* generates mutants that are able to survive heterotrophically under LAHG conditions, although they have a severe phenotype and are unable to survive in the light. In the *psaA*^*sec*^ mutants, replacement of the endogenous *psaA* gene with the *A. thaliana* homolog led to a partial complementation of the *psaA*^*prim*^ knockout phenotype (Figure 3B,D). Because this is most likely due to a partial functional substitution of the endogenous PsaA protein by its *Arabidopsis* counterpart, the degree of PSI assembly and function the *psaA*^*sec*^ mutants needs to be analysed in future experiments. Also in this case, using the single-step gene replacement strategy, both the knockout and replacement lines were generated using a single vector and a single transformation. In the case of essential genes like *psaA* the one-vector strategy confers an additional advantage because mutants with severe phenotypes can be difficult to grow in liquid cultures and therefore their transformation can be challenging. This alternative gene replacement strategy makes it possible to obtain a second recombination event simply by releasing the positive selective pressure on the segregated transformants and applying negative selection in an alternating manner.

## Conclusions

Taken together, the implementation of a double recombination event by a simple stepwise change of growth conditions can be exploited to address various biological issues and could also be employed for large-scale gene replacement approaches. Thanks to the tight counter-selection against incomplete second-round recombinants and transformants bearing multiple genomic integrations, the method could indeed be applied in partially automated systems.

## Methods

### Bacterial strains and growth conditions

The bacterial strains used are described in Table 3. *E. coli DH5*α cultures were grown in lysogeny broth (LB) medium at 37°C and shaken at 225 rpm. Unless otherwise indicated, wild-type (glucose-tolerant) and mutant *Synechocystis* sp. PCC 6803 strains were grown at 25°C and under continuous illumination at 30 μmol m^-2^ s^-1^ in BG11 medium containing 5 mM glucose [35]. Liquid cultures were shaken at 120 rpm. For growth on plates, 1.5% (w/v) agar and 0.3% (w/v) sodium thiosulfate were added to the BG11 medium. The *psaA*^*prim*^ mutant strain was grown under Light-Activated Heterotrophic Growth (LAHG) conditions (i.e. in the presence of glucose and in darkness, interrupted only by a 5-min exposure light each day) as described [27].

**Table 3:** Bacterial strains and plasmids used in this study

**Strain/plasmid**	**Characteristics**	**Selection markers**	**Source**
***E. coli***			
DH5α	Competent cells		
***Synechocystis***			
WT	WT *Synechocystis* sp PCC 6803, glucose tolerant	kan^S^, suc^R^	H. Pakrasi (Washington University, St. Louis)
*lux*^*prim*^	*luxAB* operon, interrupted by *nptI-sacB* cassette, replacing *slr0168* ORF	kan^R^, suc^S^	This study
*lux*^*sec*^	Intact *luxAB* operon replacing *slr0168* ORF	kan^S^, suc^R^	This study
*psaA*^*prim*^	*At psaA* gene, interrupted by *nptI-sacB* cassette, replacing endogenous *psaA*	kan^R^, suc^S^, heterotroph	This study
*psaA*^*sec*^	Intact *At psaA* gene replacing endogenous *psaA*	kan^S^, suc^R^, photoautotroph	This study
**Plasmids**			
pGEM-T Easy	Backbone for pDSpsaA	amp^R^	Promega, Madison, WI
pRL250	*nptI-sacB* double selection cassette, *sacB* gene from *Bacillus subtilis*	kan^R^, suc^S^	P. Wolk (Michigan State University)
pICH69822	Destination vector for Golden Gate cloning	kan^R^	E. Weber (Icon Genetics GmbH, Halle)
pRL1063a	*luxAB* operon from *Vibrio fischeri*	Sm^R^	P. Wolk (Michigan State University)
pDSlux	pICH69822 with *nptI-sacB* cassette from pRL250, *luxAB* operon from pRL1063a, *Syn psbA2* promoter and *slr0168* flanking regions	kan^R^, suc^S^	This study
pDSpsaA	pICH69822 with *nptI-sacB* cassette from pRL250, *At psaA* gene and *Syn psbA2* promoter and *slr0168* flanking regions	kan^R^, suc^S^	This study

*Abbreviations*: *SynSynechocystis*, *AtArabidopsis*, R resistant, S sensitive.

For positive selection of mutants, increasing concentrations of kanamycin (10 to 100 μg/ml) were added to the medium. For negative selection, BG11 containing 5% (w/v) sucrose was used.

### Generation of plasmids

All DNA techniques such as plasmid isolation, restriction and ligation were performed according to standard protocols [36]. *Synechocystis* sequences were obtained from Cyanobase (http://genome.kazusa.or.jp/cyanobase/Synechocystis). All the plasmids used are listed in Table 3. The fragments used in the construction of the plasmids pDSlux and pDSpsaA were amplified by PCR and purified from 1% agarose gel. Primers are listed in Additional file 2.

In case of pDSlux, the amplified segments were assembled into the final construct using the one-step Golden Gate Shuffling cloning strategy [29] and the plasmid pICH69822 as destination vector. The *Synechocystis slr0168* ORF [37] [GenBankID: 954899] was used as a neutral receptor site for stable integration of the pDSLux vector into the genome. To generate the plasmid, the genomic regions upstream and downstream of *slr0168* (~1 kbps each, HR1 and HR2 respectively) were amplified by PCR and used as flanking regions for homologous recombination. The dicistronic *luxAB* operon from *Vibrio fischeri*, derived from the pRL1063a vector [38], is composed of the *luxA* and *luxB* genes, and encodes the heterodimeric luciferase enzyme which, in presence of its substrate analogue decanal, produces a luminescent product. The operon was placed under the control of the strong *Synechocystis psbA2* promoter (P_*psbA2*_) [39] by inserting it upstream of *luxA*. Two *luxAB* amplicons were generated, the first starting at position +1 of *luxA* and ending at position +460 of *luxB* and the second starting at position +856 of *luxA* and ending at the 3’ end of *luxB*. Thus, the sequence overlap between the two fragments is 1055 base pairs long. In the final construct, the two amplicons are separated by the *nptI-sacB* double selection cassette, amplified from plasmid pRL250.

The pDSpsaA plasmid was constructed, using overlapping PCR and subsequent standard cloning steps, to replace the *Synechocystis psaA* gene with the chloroplast-encoded homolog from *Arabidopsis thaliana* (*At psaA*). To generate the vector, the *nptI-sacB* cassette was excised from the pRL250 plasmid with *BamHI* and cloned into the pGEM-T Easy vector (Promega, Madison, Wisconsin) according to the manufacturer’s instructions. The regions upstream and downstream (~500 bps each, HR1 and HR2 respectively) of the *Synechocystis psaA* gene (*slr1834*) [GenBank ID: 954060] were used as homologous sequences for gene replacement. The *At psaA* (*ArthCp022*) [GenBank ID: 844768] coding sequence was placed under the control of the endogenous *Synechocystis psaA* promoter (contained in HR1), also retaining the native intergenic region of the cyanobacterial *psaA/psaB* operon and the initial part of the native *psaB* (*slr1835*, contained in HR2) [GenBank ID: 954061]. Two fragments of *At psaA* were amplified from cDNA, the first (*5’ At psaA*) covers the stretch from +1 to +1430, the second (*3’ At psaA*) extends from +922 to the 3’ end. The two amplicons, which overlap for 509 base pairs, were assembled with the upstream and downstream flanking regions via overlapping PCR. The downstream amplicon was cloned at the 3’ end of the *nptI-sacB* cassette between the *SpeI* and *SacI* restriction sites, then the other amplicon was attached upstream of the cassette between the *ApaI* and *NotI* restriction sites.

### Transformation of *Synechocystis* and selection of first-round recombinants

*Synechocystis* WT was transformed with the vectors pDSlux and pDSpsaA. For each transformation, 10 ml of growing cells at an OD_730_ of 0.4 were harvested by centrifugation and resuspended in 1/20 volume of BG11. The cell number per ml was calculated from OD_730_ using the formula: 1 OD_730_ = 7 × 10^7^ cells, and 2 μg of plasmid DNA per transformation was added to the cells. Transformations were incubated in the light for 5 hours, and shaken the last 3 hours. After addition of fresh BG11 and overnight recovery in the dark, mutants in which the *nptI-sacB* cassette had integrated into the *slr0168* open reading frame (*lux*^*prim*^) or replaced the endogenous *psaA* gene (*psaA*^*prim*^) were positively selected on BG11 agar plates containing 10 μg/ml kanamycin. Plates bearing cells transformed with pDSlux were incubated in the light, while plated cells transformed with pDSpsaA were incubated under LAHG conditions. The number of transformants obtained was then counted and transformation efficiencies were calculated based on the number of input cells employed. To ensure complete segregation of the mutants, the kanamycin concentration was progressively increased (up to 100 μg/ml).

### Counter-selection and calculation of the frequency of second-round recombinants

For the second round of recombination and subsequent counter-selection, 10 independent *lux*^*prim*^ strains were used. One *psaA*^*prim*^ mutant was also selected. Each strain was grown to an OD_730_ of 1 in liquid BG11 containing 100 μg/ml kanamycin. A 500-μl sample was taken from each of these cultures, and cells were pelleted, washed and resuspended in 10 volumes of BG11 without antibiotic. The cell number per ml was calculated as described before. As *Synechocystis* cells contain multiple copies of the genome, liquid cultures were grown for 5 days without selection in order to allow them to lose all copies of *sacB*. Then 2-ml samples of each liquid culture were plated on BG11 solid medium containing 5% sucrose. Plates were incubated in dim light (5 μmol photons m^-2^ s^-1^) in the case of the recombinants derived from *psaA*^*prim*^, in normal light for those obtained from the *lux* mutants, and the frequency of second-round recombination was determined. Five *lux*^*prim*^ mutants, and two mutants that had undergone second-round recombination (*lux*^*sec*^) deriving from each of them, were selected and used for subsequent analyses. One second-round recombinant deriving from a *psaA*^*prim*^ mutant (*psaA*^*sec*^) was also selected and further grown under normal light conditions.

Genomic PCR was used to confirm the complete segregation of first- and second-round recombinants. Genomic DNA was extracted from *Synechocystis* using the xanthogenate method [40]. Primers used to genotype the *lux* mutants were: Lux1 FW (P1), DS_*nptI* RV (P2), *sac*_S2 (P3) and Lux2 RV (P4), *slr0168* DR FW (P5), *slr0168* DW RV (P6). The primers used for genotyping of the *psaA* mutants were: *psaA* gUR FW (P7), DS_*nptI* RV (P2), *psaA*_*Syn* RV (P8), A2 FW (P9) and A3 RV (P10).

### Southern analysis

For Southern analysis, 5 μg of genomic DNA were digested with *XmaI* endonuclease. The fragments were electrophoretically separated on a 0.8% agarose gel in 0.5× TBE (40 mM Tris–HCl, pH 8.3, 45 mM boric acid, 1 mM EDTA) and then blotted onto nylon membrane (Hybond N+; GE Healthcare, http://www.gehealthcare.com/). The *DS_lux* probe (1.7 kbps) was amplified with primers Lux1 FW and Lux1 RV. The probe was labeled using the random-priming method with ^32^P[dCTP]. Hybridization was carried out overnight at 67°C. Signals were detected with a phosphoimager (Typhoon; GE Healthcare, http://www.gehealthcare.com/).

### Luciferase assay

Luciferase activity was induced by the addition of decanal (an analogue of the luciferase substrate luciferin) to the cyanobacterial suspension to a final concentration of 1 mM (from 50 mM decanal in methanol/water 50%, v/v stock). The reaction was incubated for 15 min with mild shaking and luminescence was measured with a microplate reader (Safire^2^; Tecan, http://www.tecan.com/) at room temperature. Luminescence values are expressed relative to the optical density of the cell suspension at 730 nm, also measured with the microplate reader. Each suspension was measured in duplicate and the assay was repeated twice with independently grown cultures.

To assay luciferase activity on solid medium, the decanal solution was added to cells grown on agar plates, and luminescence was detected with the FUSION FXT imaging system (Peqlab, http://www.peqlab.com/).

### Bacterial whole-cell absorbance spectra

Absorbance spectra of whole *Synechocystis* cells were recorded using a spectrophotometer. Cells were harvested, washed and resuspended in BG11 liquid medium to a final OD_730_ of 0.5. Their absorbance spectra were recorded between 350 and 750 nm and corrected for the light scattering at 730 nm.

## Competing interests

The authors declare that they have no competing financial interests.

## Authors’ contributions

SV carried out the experimental work and drafted together with TR and DL the manuscript. All authors participated in the design of the study. DL conceived the study. All authors read and approved the final manuscript.

## Supplementary Material

Additional file 1 Schematic depiction of the classical double recombination strategy. Click here for file

Additional file 2 **List of primers used in this study.** Restriction sites are indicated by bold characters. The *BsaI*-generated sticky ends used for Golden Gate Shuffling assembly of the pDSlux constructs are underlined. In the case of the pDSpsaA plasmid, regions used for assembly with overlapping PCR are indicated in italics. Abbreviations: *Syn*, *Synechocystis*; *At*, *Arabidopsis*. Click here for file
