# Supplementary material for: A single vector-based strategy for marker-less gene replacement in Synechocystis sp. PCC 6803
Source: Microb Cell Fact. 2014 Jan 8;13:4. doi: 10.1186/1475-2859-13-4 (PMC3893515; doi:10.1186/1475-2859-13-4)
Supplement: Additional file 1 — Schematic depiction of the classical double recombination strategy. [file 1475-2859-13-4-S1.pdf]

Supplementary data

A single vector-based strategy for marker-less gene replacement in *Synechocystis* sp. PCC 6803

Stefania Viola[1], Thilo Rühle[1], Dario Leister[1,§]

[1]Department Biology I, Ludwig-Maximilians-Universität München, Großhaderner Str. 2, D-82152 Planegg-Martinsried, Germany

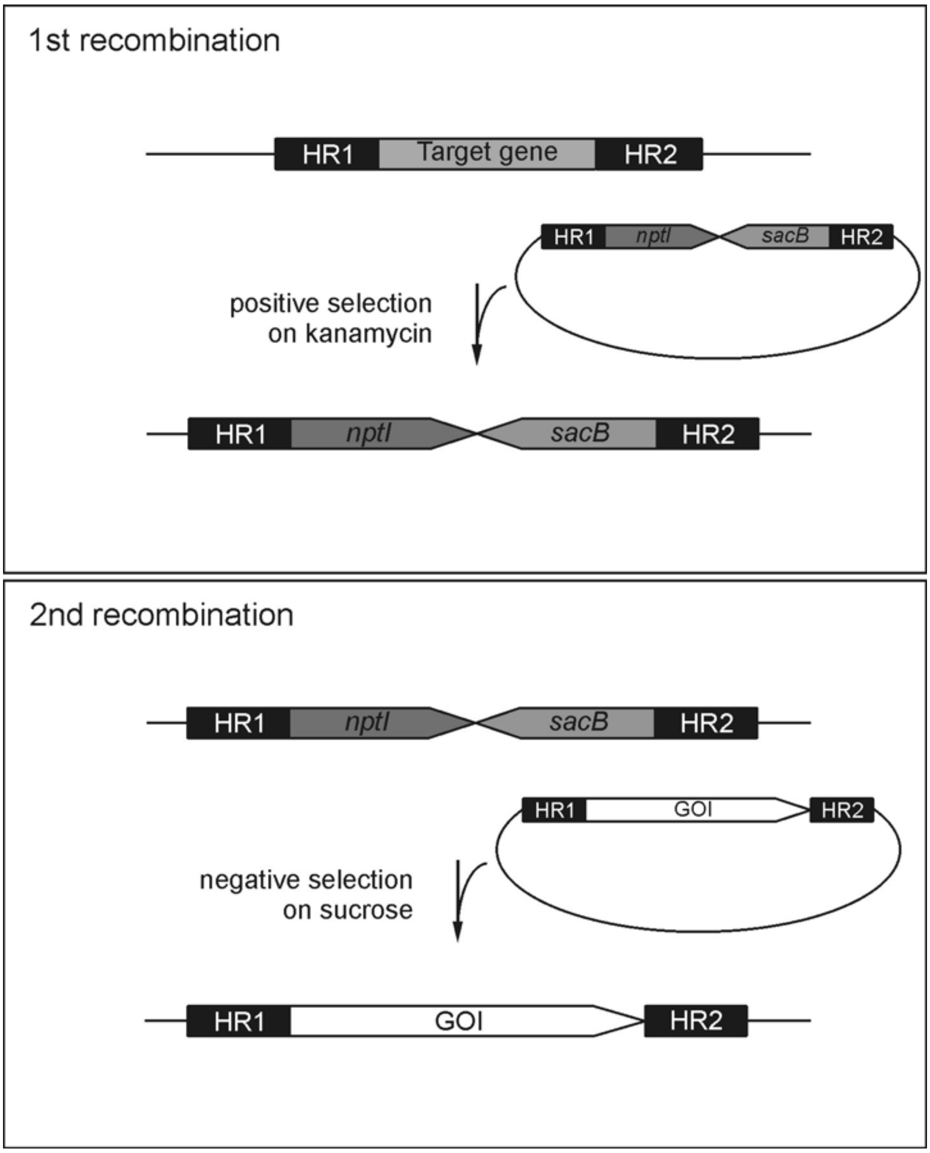

Additional File 1. Schematic depiction of the classical double recombination strategy.
