# Supplementary material for: A single vector-based strategy for marker-less gene replacement in Synechocystis sp. PCC 6803
Source: Microb Cell Fact. 2014 Jan 8;13:4. doi: 10.1186/1475-2859-13-4 (PMC3893515; doi:10.1186/1475-2859-13-4)
Supplement: Additional file 2 — List of primers used in this study. Restriction sites are indicated by bold characters. The BsaI-generated sticky ends used for Golden Gate Shuffling assembly of the pDSlux constructs are underlined. In the case of the pDSpsaA plasmid, regions used for assembly with overlapping PCR are indicated in italics. Abbreviations: Syn, Synechocystis; At, Arabidopsis. [file 1475-2859-13-4-S2.pdf]

Additional File 2. List of primers used in this study.

Primer Name	Sequence (5'→3')	Purpose	Restriction site
slr0168 UR FW	TTT**GGTCTC**TAGGTACAGGCCCTCAAGGCCCTG	PCR of *slr0168* upstream region	*BsaI*
slr0168 UR RV	TTT**GGTCTC**TGCCACTGTTATTTTGATTGGTGGC		*BsaI*
slr0168 DR FW (P5)	TTT**GGTCTC**TTTCGTTTGCGAATTTACACCAG	PCR of *slr0168* downstream region	*BsaI*
slr0168 DR RV (P6)	TTT**GGTCTC**TAAGCTAGGGTGGAGCCAGTGGC		*BsaI*
selection cassette FW	TTT**GGTCTC**ACGTTGGAATTCGATTGATCCGTCGAC	PCR of *nptI_sac*B double selection cassette	*BsaI*
selection cassette RV	TTT**GGTCTC**CCATACTTTAGGCCCGTAGTCTGCA		*BsaI*
Lux1 FW (P1)	TTT**GGTCTC**TCCAAATGAAGTTTGGAAATATTTGTTTTTC	PCR of *luxAB* first amplicon	*BsaI*
Lux1 RV	TTT**GGTCTC**TAACGCATAAAAGTCGTTTTGGGGATG		*BsaI*
Lux2 FW	TTT**GGTCTC**TTATGGTATGACTGCTGAGTCCGCAAG	PCR of *luxAB* second amplicon	*BsaI*
Lux2 RV (P4)	TTT**GGTCTC**TCGAATTGTTGAATAAATCGAACTTTTGC		*BsaI*
DS prom FW	TTT**GGTCTC**TTGGCCCCATGGAAAAAACGACAATTAC	PCR of *psabA2* promoter	*BsaI*
DS prom RV	TTT**GGTCTC**TTTGGTTATAATTCCTTATGTATTTGTCG		*BsaI*
DS_*nptI* RV (P2)	AAGATGCGTGATCTGATCCTTC	Primers used to check presence of *nptI_sacB*	
*sac*_S2 (P3)	AGCATATCATGGCGTGTAATATGGG		
A1 FW	GGCCGC**GGGCCC**GATTTCCCCTTGCGGACTCTGAGCCAATTTG	PCR of *Syn psaA* upstream region	*ApaI*
A1 RV	TGGTTCCGGCGAACGAATAAT*CATGCAGGGTTCTCCTCGCTCGACAATG*		
A2 FW (P9)	*CATTGTCGAGCGAGGAGAACCCTG*CATGATTATTCGTTCGCCGGAACCAG	PCR *At psaA* first amplicon	
A1DS RV	TTCGAAT**GCGGCCGC**TGTAATTGTATAGC		*NotI*
A4DS FW	TCAATC**ACTAGT**ATGTATAGGAC	PCR *At psaA* second amplicon	*SpeI*
A3 RV (P10)	*GGAAAACCCAACTTCAGCTCAAA*GTTTATCCTACTGCAATAATTCTTGC		
A4 FW	GCAAGAATTATTGCAGTAGGATAAAC*TTTGAGCTGAAGTTGGGTTTTCC*	PCR of *Syn psaA* downstream region	
A4 RV	GGCCGC**GAGCTC**CGATCGGGCGAATGTTTAAAGGATCTTTAATC		*SacI*
psaA gUR FW (P7)	TGTGAGGGAACTTGGAACTC	Primers used to check presence of *Syn psaA*	
psaA_syn RV (P8)	GATCATAATCACGCACCATG		

Restriction sites are indicated by bold characters. The *BsaI*-generated sticky ends used for Golden Gate Shuffling assembly of the pDSlux constructs are underlined. In the case of the pDSpsaA plasmid, regions used for assembly with overlapping PCR are indicated in italics. Abbreviations: *Syn*, *Synechocystis*; *At*, *Arabidopsis*.
